# A Novel Yeast Genus and Two Novel Species Isolated from Pineapple Leaves in Thailand: *Savitreella phatthalungensis* gen. nov., sp. nov. and *Goffeauzyma siamensis* sp. nov.

**DOI:** 10.3390/jof8020118

**Published:** 2022-01-26

**Authors:** Pumin Nutaratat, Wanatchaporn Boontham, Pannida Khunnamwong

**Affiliations:** 1 Department of Biology, Faculty of Science, Thaksin University, Pa Phayom, Phatthalung 93210, Thailand; pumin.n@tsu.ac.th; 2 Microbial Technology for Agriculture, Food and Environment Research Center, Faculty of Science, Thaksin University, Pa Phayom, Phatthalung 93210, Thailand; 3 Program of Microbiology, Faculty of Science and Technology, Nakhon Pathom Rajabhat University, Nakhon Pathom 73000, Thailand; wanatchaporn@webmail.npru.ac.th; 4 Department of Microbiology, Faculty of Science, Kasetsart University, Bangkok 10900, Thailand; 5 Biodiversity Center, Kasetsart University (BDCKU), Bangkok 10900, Thailand

**Keywords:** two new taxa, yeast taxonomy, yeast phylogeny, *Savitreella phatthalungensis*, *Goffeauzyma siamensis*, pineapple, phyllosphere

## Abstract

Four yeast strains, representing one genus and two novel anamorphic yeast species, were isolated from pineapple leaves collected in Thailand. Analysis of the sequences of the D1/D2 domains of the large subunit (LSU) rRNA gene and the internal transcribed spacer (ITS) regions showed the two strains (DMKU-PAL186 and DMKU-PAL178) were closely related to the type strains of the *Protomyces* and *Taphrina* species, but with high nucleotide divergence. Two strains (DMKU-PAL39 and DMKU-PAL18) were found to be closely related to the type strains of *Goffeauzyma iberica*, but with eight nucleotide substitutions in the D1/D2 domains and 26 nucleotide substitutions in the ITS regions. In phylogenetic analyses, the strains DMKU-PAL186 and DMKU-PAL178 formed a well-separated lineage from *Protomyces* and *Taphrina* genera, confirming that they represented a distinct genus, while the strains DMKU-PAL39 and DMKU-PAL18 represented a species in the genus *Goffeauzyma*, which was phylogenetically distinct from other recognized species of the genus. Based on molecular analyses and phenotypic characteristics, the names *Savitreella* gen. nov. (Taphrinomycetes, Ascomycota) and *Savitreella phatthalungensis* sp. nov. are proposed to accommodate the strains DMKU-PAL186 and DMKU-PAL178, and the name *Goffeauzyma siamensis* sp. nov. (Tremellomycetes, Basidiomycota) is proposed to accommodate the strains DMKU-PAL39 and DMKU-PAL18.

## 1. Introduction

Pineapple (*Ananas comosus*), belonging to the family Bromeliaceae, is a well-known and readily available tropical fruit. The global production of pineapple is projected to grow at 1.9 percent annually, to reach 31 million tonnes in 2028 [1]. In Thailand, the pineapple is one of the most important plants to be grown commercially. Pineapple is a source of functional ingredients (fiber) [2], anti-inflammatory agents (bromelain) [3], antioxidants (flavonoids and phenolic acids), and vitamins (vitamin C) [4]. It has a high sugar content (sucrose, glucose, and fructose) [5] and is consequently the habitat of several microorganisms, including lactic acid bacteria (i.e., *Lactobacillus plantarum* and *L. rossiae*) and yeasts (i.e., *Meyerozyma caribbica* and *Pichia guilliermondii*) [6,7]. However, there are only a few reports of yeast being associated with the pineapple phyllosphere. Pineapple leaves exhibit an extreme environment involving such factors as high temperature, variable relative humidity, radiation, and acidic conditions [8]. Therefore, microorganisms that can grow on pineapple leaves need to be adapted to survive under variable and severe conditions.

Genera *Taphrina* and *Protomyces* are classified in the families Taphrinaceae and Protomycetaceae, respectively, which in turn are members of the order Taphrinales (subphylum Taphrinomycotina, phylum Ascomycota). Members of these two genera display varying color colonies with a reddish, salmon-like color, lighter shades of red, lavender, or yellow, that arise from the presence of carotenoids [9]. The genus *Protomyces* was first established, as an anamorphic genus, by Unger in 1833, and *P. macrosporus* was assigned as a type species of the genus. This has been strictly defined, based morphologically on cell sizes and their host range (Compositae and Umbelliferae) [9,10]. Later, Wang et al. [11] detected *Protomyces* species from a plant outside of its host families (Compositae and Umbelliferae), indicating the genus *Protomyces* may not be strictly associated with only the Compositae or Umbelliferae family host plants. Recently, an additional member of *Protomyces,* namely, *P. arabidopsidicola*, was found from the phylloplane of *Arabidopsis thaliana* (Brassicaceae) [12].

The genus *Taphrina* was created by Fries in 1832 [13] to accommodate the teleomorphic yeast state in which *T. populina* was assigned as a type species of the genus. This genus has a dimorphic lifestyle, where the teleomorph is the parasitic counterpart on plants and the anamorphic yeast state represents the saprotrophic state, which is classified in the genus *Lalaria*. The genus *Taphrina* has been defined as being based on the host range, geographical distribution, morphological features (i.e., the size and shape of asci and ascospores), the type and site of infection, and their ability to cause specific infections on host plants [14]. However, the validity of species separation on related hosts has been debated by several authors [15]. In 2003, Rodrigues and Fonseca [16] studied the *Taphrina* species based on the sequence analysis of the D1/D2 domains of the LSU rRNA gene and ITS regions and confirmed that the genus *Taphrina* was distinct from its closely related genus *Protomyces*. Later, Selbmann et al. [17] identified yeasts isolated from sandstone and granite and proposed as a new member of the genus *Taphrina*, namely, *Taphrina antarctica,* based on analysis of the ITS regions and the D1/D2 domains of the LSU rRNA gene. In addition, the reclassification of four *Lalaria* species (*La. arrabidae*, *La. inositophila*, *La. kurtzmanii,* and *La. veronaerambellii*) is also proposed as novel combinations of the genus *Taphrina*, according to the *International Code of Nomenclature for Algae, Fungi, and Plants* (Melbourne Code, 2012). This report suggested that the genus *Taphrina* may not be strictly associated only with host plants, and the use of molecular tools for species identification in fungi allows for an easy connection between teleomorphic and anamorphic states, even when the morphology of the two forms differs considerably [17].

The genus *Goffeauzyma* was first proposed in 2015 by Liu et al. [18], based on the phylogenetic analysis of a seven-gene dataset consisting of the small subunit (SSU) rRNA gene, the internal transcribed spacer (ITS) regions of the rRNA gene, the D1/D2 domains of the large subunit (LSU) rRNA gene, the two subunits of RNA polymerase II (*RPB1* and *RPB2*), the translation elongation factor 1-alpha (*TEF1*) and cytochrome b (*CYTB*). In the seven-gene dataset and the rDNA phylogeny, this genus represented a well-supported clade containing six *Cryptococcus* species, which was formerly recognized as the *gastricus* clade [19], namely, *C. aciditolerans*, *C. ibericus*, *C. metallitolerans*, *C. agrionensis*, *C. gastricus*, and *C. gilvescens* [20,21,22]. The gastricus clade was located in the family Filobasidiaceae (subphylum Agaricomycotina and phylum Basidiomycota) but was phylogenetically-distinguished from the recognized species in the genera *Filobasidium*, *Naganishia,* and *Heterocephalacria* [18]. According to the Melbourne Code [23], which specifies that related anamorphic and teleomorphic species can be assigned to the same genus, numerous anamorphic species were consequently reassigned to the teleomorphic genera, based on their phylogenetic relationship, and many novel anamorphic genera were proposed. In view of the above, the genus *Goffeauzyma* was also established to accommodate the six *Cryptococcus* species of the gastricus clade, which were then renamed as *G. aciditolerans*, *G. agrionensis*, *G. gastricus*, and *G. gilvescens*, *G. ibericus,* and *G. metallitolerans*, respectively. Of these, *G. gastricus* was designated as the type species of the genus [18].

In the present study, four strains (DMKU-PAL186, DMKU-PAL178, DMKU-PAL39, and DMKU-PAL18) representing a novel genus and two species, namely, *Savitreella phatthalungensis* gen., sp. nov. (DMKU-PAL186 and DMKU-PAL178) and *Goffeauzyma siamensis* sp. nov. (DMKU-PAL39 and DMKU-PAL18) were proposed based on a polyphasic taxonomy.

## 2. Materials and Methods

### 2.1. Sample Collection and Yeast Isolation

Green and healthy leaves of pineapple (*Ananas comosus*) were randomly collected from cultivated fields in Chon Buri province (21 samples), eastern region of Thailand, on December 26, 2020, and Phatthalung province (10 samples) southern region of Thailand, on January 13, 2021. Leaf samples (five leaves per sample) were put in plastic bags, sealed, and kept in icebox for 6–12 h during transfer to the laboratory. The samples were stored at 8 °C until subjected to yeast isolation, which was no longer than 48 h.

The yeasts were isolated by a dilution plate technique from the healthy pineapple leaves. The leaves (5 g) were suspended in 50 mL of 0.85% saline solution in a 250 mL Erlenmeyer flask and shaken on a rotary shaker at 150 rpm, 25 °C for 60 min to detach yeast cells from the surface. The suspension was serially diluted tenfold (1:10 to 1:10^3^) by sterile 0.85% (*w/v*) saline solution; then, 100 µL of each dilution was spread onto yeast extract-malt extract (YM) agar (0.3% (*w/v*) yeast extract, 0.3% (*w/v*) malt extract, 0.5% (*w/v*) peptone, 1% (*w/v*) glucose, and 2% (*w/v*) agar) supplemented with 0.02% (*w/v*) chloramphenicol and incubated at 25 °C for seven days. The different yeast morphotypes were picked and purified by streaking on YM agar. All purified yeast strains were suspended in YM broth supplemented with 10% (*v/v*) glycerol and stored at −80 °C.

### 2.2. Yeast Identification

#### 2.2.1. Morphological Study

The morphological characteristics of yeast strains were determined according to established methods by Kurtzman et al. [24]. Colony characters were observed on YM agar after two days of incubation in darkness at 25 °C. Mycelium formation was investigated by cultivation on potato dextrose agar (PDA; 20% (*w/v*) potato infusion, 2% (*w/v*) glucose, and 1.5% (*w/v*) agar) in slide culture at 25 °C for four weeks. Sexual processes of all strains were investigated for individual strain and strain pairs on PDA, corn meal agar (2% (*w/v*) cornmeal infusion and 2% (*w/v*) agar), 5% malt extract agar (5% (*w/v*) malt extract and 1.5% (*w/v*) agar), Fowell’s acetate agar (0.5% (*w/v*) sodium acetate and 2% (*w/v*) agar), Gorodkowa agar (0.1% (*w/v*) glucose, 0.5% (*w/v*) sodium chloride, 1% (*w/v*) peptone and 2% (*w/v*) agar), and V8 agar (10% (*v/v*) V8 juice and 2% (*w/v*) agar) at 15 °C and 25 °C for two months.

#### 2.2.2. Biochemical and Physiological Studies

Four strains (DMKU-PAL186, DMKU-PAL178, DMKU-PAL39, and DMKU-PAL18) were characterized biochemically and physiologically, according to the standard methods described by Kurtzman et al. [24]. Fermentation of carbohydrates was carried out in a liquid medium using Durham fermentation tubes. Carbon source and nitrogen source assimilation tests were conducted in liquid medium and starved inocula were used in nitrogen assimilation tests [24]. Cycloheximide resistance was also performed in a liquid medium, while urea hydrolysis was conducted on agar slants. Acid production and the diazonium blue B (DBB) reaction were investigated on a solid medium in Petri dishes. Growth at various temperatures (15, 30, 35, 37, and 40 °C) was determined by cultivation on YM agar.

#### 2.2.3. Molecular Study

Each yeast strain was grown on YM agar for two days. The target gene sequences were determined from polymerase chain reaction (PCR) products amplified from genomic DNA extracted from yeast cells. The methods used for DNA extraction and amplification were as described by Limtong et al. [25]. The D1/D2 domains of the LSU rRNA gene, ITS regions, SSU rRNA gene, *TEF1*, and *RPB2* genes were amplified with the primer pairs NL1 and NL4 (for D1/D2 domains) [26], ITS1 and ITS4 (for ITS regions) [27], SSU1f and SSU4r, SSU3f and SSU2r (for SSU) [28], EF1-983f and EF1-2218r (for *TEF1*) [29], and RBP2-5f and RPB2-7r (for *RPB2*) [30]. The PCR products were checked by agarose gel electrophoresis and purified using the TIANgel Midi Purification kit (TIANGEN Biotech), according to the manufacturer’s protocol. The purified PCR product was sequenced with the same primers used in PCR amplification at the First BASE Laboratories Sdn Bhd, Malaysia. The newly generated sequences were submitted to GenBank (http://www.ncbi.nlm.nih.gov/genbank, accessed on 30 November 2021) and the GenBank accession number of the sequences used in this study are shown in Table 1 and Table 2.

Sequences generated from the forward and reverse primers were aligned and assembled with MEGA software, version 11 (MEGA11) [31], to get full length of the gene sequence. Then, the assembled sequences were compared with the GenBank database using the BLASTn search tool [32]. Multiple sequence alignment was performed using the MUSCLE algorithm. Phylogenetic analyses, based on the concatenated sequences of the ITS regions and the D1/D2 domains of the LSU rRNA gene and the multilocus datasets, using the neighbor-joining (NJ) and the maximum likelihood (ML) methods were performed with MEGA11 software. The Kimura-2 parameter distance correction and the general time reversible (GTR) models were respectively used for the NJ and ML analyses. Confidence levels of the clades were estimated from bootstrap analysis (1000 replicates) [33].

## 3. Results

### 3.1. Yeast Isolation

Strains DMKU-PAL186 and DMKU-PAL178 were respectively isolated from two pineapple leaf samples, one leaf sample collected at sampling site No. 7 (7°14′5″ N/100°10′54″ E) and another leaf sample at sampling site No. 9, (7°15′56″ N/100°10′23″ E), and both located in Phatthalung province, while strains DMKU-PAL39 and DMKU-PAL18 were isolated from the healthy pineapple leaf samples collected from two different cultivated fields (13°06′35.6″ N/101°06′16.9″ E, for DMKU-PAL39 and 13°11′48.9″ N/100°59′37.6″ E, for DMKU-PAL18) located in Chon Buri province.

During the investigation of yeasts associated with pineapple leaves collected from cultivated fields in Thailand, two yeast strains (DMKU-PAL186 and DMKU-PAL178) representing a novel yeast taxon of the Taphrinomycetes, Ascomycota were discovered. These two strains were found to be closely related to the type strains of the *Protomyces* and *Taphrina* species. In addition, another two strains (DMKU-PAL39 and DMKU-PAL18) representing a novel species of the genus *Goffeauzyma* (Tremellomycetes, Basidiomycota) were also obtained.

### 3.2. Molecular Analyses and Phenotypic Characterization

#### 3.2.1. Strain DMKU-PAL186 and DMKU-PAL178

Analysis of the sequences of the D1/D2 domains of the LSU rRNA gene and ITS regions demonstrated that the strains DMKU-PAL186 and DMKU-PAL178 were identical. BLASTn searches of the GenBank database revealed that the D1/D2 sequence of the two strains DMKU-PAL186^T^ and DMKU-PAL178 are closely related to the type strains of *Protomyces*, whereas the ITS sequences of these two strains are closely related to the type strains of the *Taphrina* species, followed by the *Protomyces* species. Consequently, all *Protomyces* species (*P. inouyei, P. pachydermus, P. lactucaedebilis, P. macrosporus, P. gravidus*, *P. inundatus,* and *P. arabidopsidicola*) and six of the *Taphrina* species (*T. virginica*, *T. wiesneri*, *T. letifera*, *T. communis*, *T. deformans,* and *T. carnea*) showing a high degree of similarity in the BLAST searches were selected to compare genetic divergences. In terms of pairwise sequence similarities, the two strains (DMKU-PAL186 and DMKU-PAL178) differed from the type strains of the *Protomyces* species by 52–66 nucleotide (nt) substitutions in the D1/D2 domains and differed from a group of the related *Taphrina* species, by 63–65 nt substitutions in the D1/D2 domains (Table 3). In addition, the ITS sequences of the two strains differed from the *Protomyces* species and the *Taphrina* species by 57–106 and 89–114 nt substitutions, respectively (Table 3).

**Figure 1:**
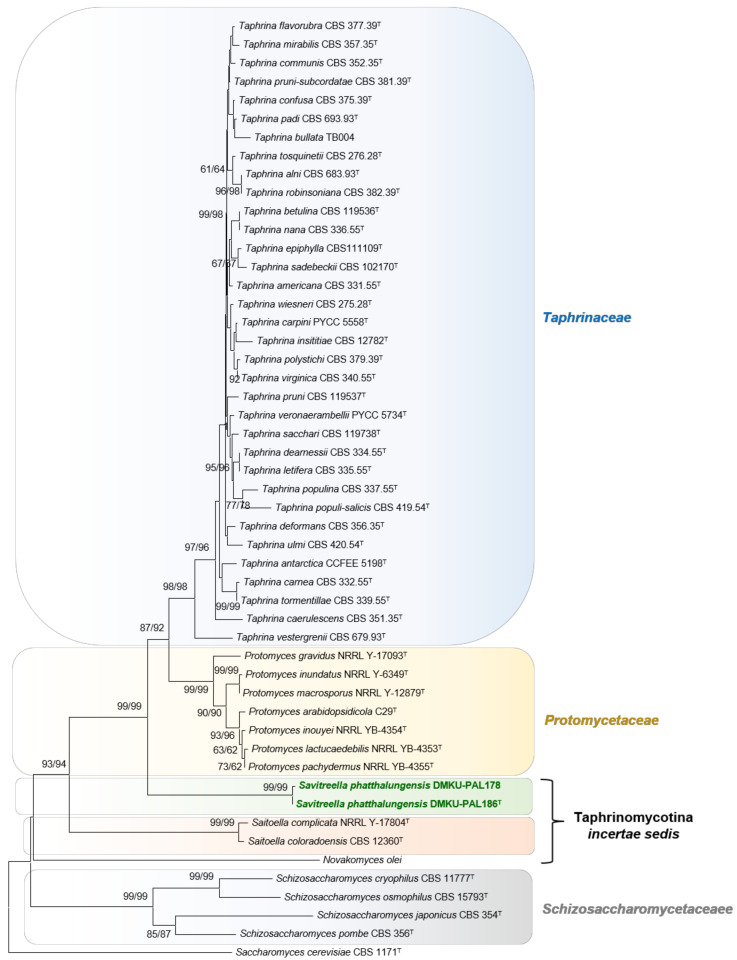
Phylogenetic tree based on the combined sequences of the ITS regions and the D1/D2 domains of the LSU rRNA gene, showing positions of *Savitreella phatthalungensis* sp. nov. with respect to closely related species. The tree backbone was constructed with the neighbor-joining method by MEGA software. Numbers at the node indicate percentages of bootstrap sampling (BP) of neighbor-joining (NJ) and maximum likelihood (ML) analyses, derived from 1000 samples. All positions containing gaps and missing data were eliminated, resulting in a total of 731 positions in the final dataset. *Saccharomyces cerevisiae* CBS 1171^T^ was used as outgroup in these analyses. Bar, patristic distance of 0.05.

**Figure 2:**
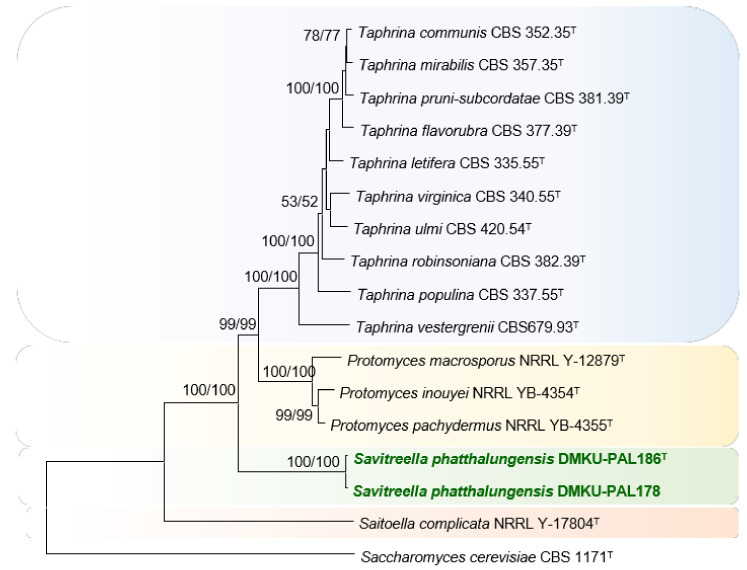
Phylogenetic tree based on the combined sequences of the SSU rRNA gene, ITS regions and the D1/D2 domains of the LSU rRNA gene, showing positions of *Savitreella phatthalungensis* sp. nov. with respect to closely related species. The tree backbone was constructed with the neighbor-joining method by MEGA software. Numbers at the node indicate percentages of bootstrap sampling (BP) of the NJ and ML analyses, derived from 1000 samples. All positions containing gaps and missing data were eliminated, resulting in a total of 4392 positions in the final dataset. *Saccharomyces cerevisiae* CBS 1171^T^ was used as outgroup in these analyses. *Bar*, patristic distance of 0.05.

In phylogenetic analysis based on the concatenated sequences of the ITS and the D1/D2 domains of the LSU rRNA gene, the trees derived from neighbor-joining (NJ) and maximum likelihood (ML) analysis were found to be similar and showed that the two strains formed a monophyletic clade closely related to *Protomyces* and *Taphrina* species, with strong statistical support (Figure 1). To clarify the placement of the two strains, the phylogenetic analysis of a multilocus dataset (SSU, ITS, LSU, *TEF1*, *ACT1,* and *RPB2*) needs to be conducted. However, the sequence data of the other marker genes (e.g., *RPB2*, *TEF1,* and *ACT1*) of several *Protomyces* and *Taphrina* species are not available in the public online databases. Consequently, the phylogenetic analysis based on the combined sequences of the SSU, ITS, and the D1/D2 domains of the two strains, and the type strains of representative species in the Taphrinomycotina with available SSU rRNA data, were performed in this study. From these analyses, the NJ and ML trees made it clear that the two strains formed a well-separated lineage from the neighboring genera, *Protomyces* and *Taphrina* (Figure 2), and confirmed the presence of the monophyletic clade that was phylogenetically distinct from any recognized families with yeast state in the Taphrinomycotina (Protomycetaceae, Schizosaccharomycetaceae, and Taphrinaceae) as well as two recognized genera in the Taphrinomycotina *incertae sedis* (*Saitoella* and *Novakomyces*), providing deeper level support (100% bootstrap support) for considering them as a distinct species and genus (Figure 1 and Figure 2). Since the family assignment of the Taphrinomycotina is still unclear, we chose not to assign the family of the new genus and have left open the family assignment until the existing family structure within the Taphrinomycotina is better clarified, based on eventual phylogenomic data.

**Figure 3:**
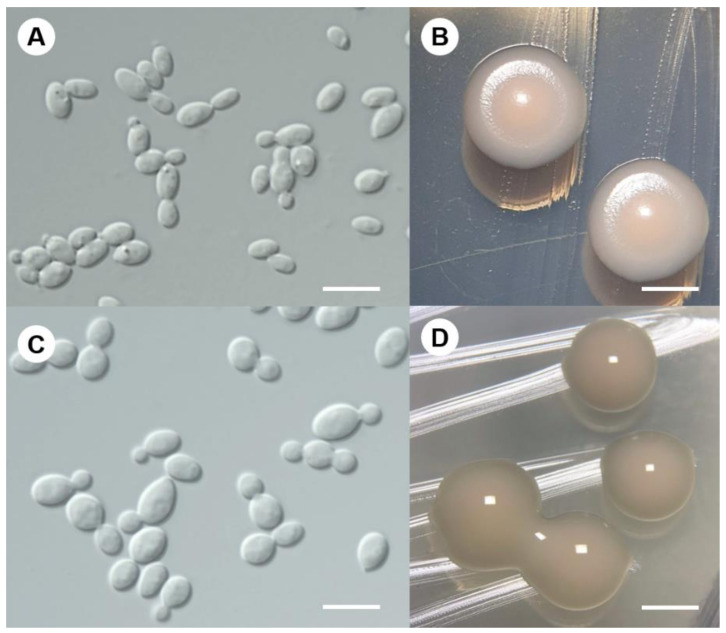
Budding cells and colony morphology on YM agar after 3 days at 25 °C. (**A**,**B**) *Savitreella phatthalungensis* (holotype DMKU-PAL186); (**C**,**D**) *Goffeauzyma siamensis* (holotype DMKU-PAL39). Scale bars: (**A**,**C**) = 10 µm; (**B**,**D**) = 100 µm.

In the comparison between morphological and phenotypic characteristics of the proposed novel species and type strains of the related species in genera *Protomyces* and *Taphrina*, we found the colonies of the strain DMKU-PAL186^T^ on YM agar are cream to light pinkish–red, convex and with entire margins (Figure 3B), which is consistent with the colony color of *Protomyces* species reported by Kurtzman [9] and Wang et al. [12]. However, these two strains can be distinguished from their phylogenetically closest recognized neighbors by some phenotypic characteristics, as shown in Table 4. The proposed novel species assimilates D-gluconate, while the related *Protomyces* and *Taphrina* species do not. Several *Protomyces* and *Taphrina* species assimilate D-arabinose, L-arabinose, and potassium nitrate, but the proposed novel species does not. Starch formation is negative for the proposed novel species but is positive for the *Protomyces* (weak positive) and *Taphrina* species. Growth at 30 °C is present for the proposed novel species but is absent for the related *Protomyces* and *Taphrina* species. Based on polyphasic analyses (genetic divergent, phylogenetic, and phenotypic characteristics), we concluded that these two strains, DMKU-PAL186 and DMKU-PAL178, represent a novel genus and species of the subphylum Taphrinomycotina. The name *Savitreella phatthalungensis* gen. nov., sp. nov. is proposed to accommodate these yeast strains.

Data for species 1 is from the present study, for species 2–7 and 9–15 from Kurtzman [9] and for species 8 from Wang et al. [12].

#### 3.2.2. DMKU-PAL39 and DMKU-PAL18

The sequence analyses of the D1/D2 domains of the LSU rRNA gene and ITS regions revealed that the two strains (DMKU-PAL39 and DMKU-PAL18) had identical sequences, indicating that the two strains represented a single species. Pairwise sequence comparison of the D1/D2 domains and ITS regions of strain DMKU-PAL39 with respect to its related species showed that these strains were distinct from their closest species, *Goffeauzyma iberica* CBS 10871^T^, which differed by 8 nt substitutions (1.4%) of 570 nt in the D1/D2 domains and 26 nt substitutions of 454 nt (6.2%) in the ITS regions.

**Figure 4:**
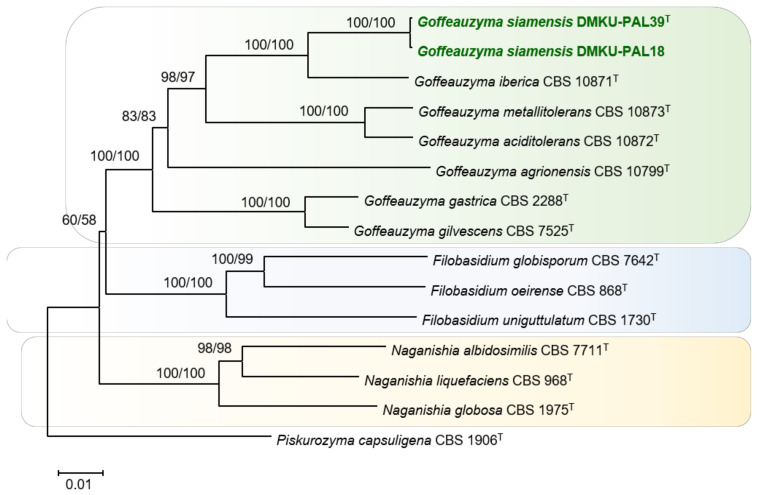
Phylogenetic tree of *Goffeauzyma siamensis* sp. nov. based on the concatenated sequences of the SSU rRNA, ITS regions, D1/D2 domains of the LSU rRNA, *RPB2* and *TEF1* genes showing positions of the three strains of a novel species with respect to closely related species. The tree backbone was constructed with the neighbor-joining method by MEGA software. Numbers at the node indicate percentages of bootstrap sampling (BP) of the NJ and ML analyses, derived from 1000 samples. All positions containing gaps and missing data were eliminated, resulting in a total of 2123 positions in the final dataset. *Piskurozyma capsuligena* CBS 1906^T^ was used as outgroup in these analyses. Bar, patristic distance of 0.01.

A phylogenetic analysis based on the combined sequences of the ITS regions and the D1/D2 domains of the LSU rRNA gene demonstrated that the strains DMKU-PAL39 and DMKU-PAL18 placed in the *Goffeauzyma* species, but in a distinct position from the other *Goffeauzyma* species in the clade, with relatively high bootstrap support (Figure 4). To confirm the placement of these two strains, phylogenetic analyses of the combined sequences of the SSU rRNA, ITS regions, D1/D2 domains of the LSU rRNA, *RPB2* and *TEF* genes of these two strains and the type strains of the *Goffeauzyma* species were performed by using the NJ and ML methods. From these analyses, the NJ and ML trees were found to be similar, making it clear that the two strains were placed in the *Goffeauzyma* clade and formed a well-separated lineage closely related to *Goffeauzyma iberica* (Figure 4).

In practice, *Goffeauzyma siamensis* nov. is not only distinguishable on the basis of molecular analyses but also by some phenotypic characteristics and growth abilities (Table 5). *G. siamensis* sp. nov. assimilates glycerol, ribitol, D-gluconate and DL-lactate, whereas *G. iberica* does not. Growth on medium containing 0.01% cycloheximide is absent for *G. siamensis* but is present for *G. iberica*. Based on this evidence, we therefore concluded that the strains DMKU-PAL39 and DMKU-PAL18 represent a single novel species of the genus *Goffeauzyma*. The name *Goffeauzyma siamensis* sp. nov. is proposed to accommodate these yeast strains.

Data for species *G. siamensis* from this study and for *G. iberica* is from Gadanho and Sampaio [20].

### 3.3. Taxonomic Description of Genus and New Species

#### 3.3.1. *Savitreella* P. Nutaratat, W. Boontham & P. Khunnamwong, gen. nov.

MycoBank: MB 840873.

Type species: *Savitreella phatthalungensis*

Etymology: *Savitreella* (Sa.vi.tre.el.la. N.L. fem. n. *Savitreella*, a yeast genus named in honor of Professor Savitree Limtong for her outstanding studies of yeast systematics and yeast technology in Thailand).

Description: The genus can be separated from the neighboring genera, *Protomyces* and *Taphrina,* by phylogenetic analyses (Figure 1 and Figure 2) and phenotypic characteristics (Table 4). Yeast cells divide by multilateral budding. Pseudohyphae are not produced. Ascospores have not been observed in individual or in mixed cultures. Glucose is not fermented. Diazonium blue B reaction, urease activity and acid production are negative. The genus is phylogenetically related to *Protomyces* and *Taphrina*.

Classification: Taphrinales, Taphrinomycetes, Taphrinomycotina, Ascomycota.

#### 3.3.2. *Savitreella phatthalungensis* P. Nutaratat, W. Boontham & P. Khunnamwong, sp. nov.

MycoBank: MB 840874.

Etymology: *Savitreella phatthalungensis* (phat.tha.lung. en’sis. N.L. fem. adj. *phatthalungensis*) refers to Phatthalung province, Thailand, where the type strain was isolated.

Holotype: DMKU-PAL186 ^T^ is the holotype of *Savitreella phatthalungensis.* It was isolated from a pineapple leaf collected from pineapple cultivated field, Phatthalung province, Thailand. It has been preserved as a metabolically inactive state in the culture collection of the Department of Microbiology, Faculty of Science, Kasetsart University, Bangkok, Thailand (DMKU). The culture ex-type has been deposited in a metabolically inactivate state in the Thailand Bioresource Research Center (TBRC), National Center for Genetic Engineering and Biotechnology, Thailand as TBRC 15109 and the Portuguese Yeast Culture Collection (PYCC), Portugal as PYCC 9005.

Description: After 3 days at 25 °C on YM agar, cells are globose (2.8 − 4.9 × 3.1 − 5.8 µm) and occur singly or in pairs (Figure 3A). Budding is predominately polar. Colonies are cream to light pinkish–red, convex, and have an entire margin (Figure 3B). Pseudohyphae or true hyphae are not formed in slide culture on PDA and corn meal agar within 4 weeks at 25 °C. Ascospores formation is not observed in any of the strains or when strains are paired on PDA, corn meal agar, 5% malt extract agar, YPD agar, YCBY agar, YM agar, and water agar at 15 and 25 °C for one month. Fermentation is absent. Glucose, xylose (or slow), sucrose, maltose, trehalose (or weak), cellobiose (slow), salicin (weak), melezitose, glycerol, D-mannitol (or weak), D-glucono-1,5-lactone, gluconate, DL-lactate (or slow), succinate, and xylitol are assimilated, but galactose, L-sorbose, *N*-acetyl glucosamine, D-ribose, L-arabinose, D-arabinose, rhamnose, methyl α-D-glucoside, melibiose, lactose, raffinose, erythritol, arabitol, D-glucitol, *myo*-inositol, glucuronate, galacturonic acid, and citrate are not assimilated. Ammonium sulfate is assimilated, but nitrate, nitrite, ethylamine hydrochloride, L-lysine, and cadaverine are not assimilated. No growth occurs on media containing 10% (*w/v*) NaCl/5% (*w/v*) glucose, 16% (*w/v*) NaCl/5% (*w/v*) glucose, 50% (*w/v*) glucose, 60% (*w/v*) glucose, and in vitamin-free medium, 0.01% cycloheximide and 0.1% cycloheximide. Growth occurs at 15–35 °C, but not at 37 °C. Starch-like compounds are not produced. Diazomium blue B color and urease reaction are negative.

GenBank accession numbers: holotype DMKU-PAL186 (SSU: LC647808, ITS: MW876306, D1/D2: MW879743); additional strain DMKU-PAL178 (SSU: LC647809, ITS: MW876305, D1/D2: MW879742).

#### 3.3.3. *Goffeauzyma siamensis* P. Nutaratat, W. Boontham & P. Khunnamwong, sp. nov.

MycoBank: MB 840692.

Etymology: The species epithet *si.am.en′sis.* N.L. fem. adj. *siamensis*, referring to Siam, the old name of Thailand, where the type strain was isolated.

Holotype: DMKU-PAL39^T^ is the holotype of *G. siamensis.* It was isolated from a pineapple leaf collected from pineapple cultivated field, Si Racha district, Chon Buri province, Thailand. It has been preserved as a metabolically inactive state in the culture collection of the Department of Microbiology, Faculty of Science, Kasetsart University, Bangkok, Thailand (DMKU). The culture ex-type has been deposited in a metabolically inactivate state in the Thailand Bioresource Research Center (TBRC), National Center for Genetic Engineering and Biotechnology, Thailand as TBRC 14871 and in the Portuguese Yeast Culture Collection (PYCC), Portugal as PYCC 8910.

Description: After 3 days at 25 °C on YM agar, cells are subglobose to ovoid (2.8 − 4.9 × 3.1 − 5.8 µm) and occur singly or in pairs (Figure 3C). Colonies are very mucoid bright cream to light brown, convex and have an entire margin (Figure 3D). Budding is polar. Pseudohyphae or true hyphae are not formed. Ballistoconidia are not produced on PDA or corn meal agar. Sexual reproduction is not observed in any of the strains or when strains are paired on PDA, corn meal agar, 5% malt extract agar, YCBY agar, V8 and YM agar at 15 and 25 °C for 2 months. Fermentation of glucose is absent. Glucose, galactose, sorbose (variable), *N*-acetyl glucosamine (variable), D-ribose (variable), D-xylose, L-arabinose, D-arabinose (variable), L-rhamnose, sucrose, maltose, trehalose, methyl α-D-glucoside, cellobiose, salicin, melibiose, lactose, raffinose, melizitose, glycerol, erythritol, ribitol, D-glucitol, D-mannitol, galactitol (week), *myo*-inositol, D-glucono-1,5-lactone, D-gluconate (slow), D-glucuronate, D-galacturonic acid, DL-lactate, succinate and xylitol are assimilated as sole carbon source, but inulin, soluble starch, 2-ketogluconic acid, 5-ketogluconic acid, citrate, methanol, and ethanol are not assimilated. Ammonium sulfate and L-lysine are assimilated as sole nitrogen source, but nitrate, nitrite, cadaverine, and creatine are not assimilated. Growth in vitamin-free medium is negative. Growth is observed at 15–37 °C but not at 40 °C. Growth in the presence of 0.01 or 0.1% of cycloheximide is negative. Growth on media containing 50% (*w/v*) glucose and 10% (*w/v*) sodium chloride plus 5% (*w/v*) glucose are positive, but on media, a containing 60% (*w/v*) glucose is weak. No growth occurred in media containing 16% (*w/v*) sodium chloride plus 5% (*w/v*) glucose. Acid production is negative. Hydrolysis of urea and DBB reaction are positive. Starch-like compounds are not produced.

GenBank accession numbers: holotype DMKU-PAL39 (SSU: OK576181, ITS: MW669577, D1/D2: LC604627 *RPB2*: LC656481, *TEF1*: LC656484); additional strain DMKU-PAL18 (SSU: OK576179, ITS: MZ621116, D1/D2: MZ621115, *RPB2*: LC656482, *TEF1*: LC656483).

## 4. Discussion

In accordance with the guidelines for ascomycetous yeast identification based on nucleotide sequence divergences, the two compared strains with 0–3 nucleotide differences in the D1/D2 domains are designated to be the same species, whereas the strains that differed by greater than 6 nucleotides (1%) are recognized as different species [26]. For basidiomycetous yeasts, the strains that differed by two or more nucleotides in the D1/D2 regions represent different taxa [34]. Later, Vu et al. [35] proposed the threshold to consider a strain to belong to a different species from its close relatives is of less than 98.31% similarity (Ascomycota) or 98.61% similarity (Basidiomycota) in terms of ITS region and less than 99.41% similarity (Ascomycota) or 99.51% similarity (Basidiomycota) when considering the D1/D2 domains. In the present study, the sequences of the D1/D2 of the LSU rRNA gene and ITS regions of the four strains were compared with their closely related species. Two strains (DMKU-PAL186 and DMKU-PAL178) were identical in the D1/D2 and ITS regions but differed from the type strains of the closely related species by 52–66 nucleotide substitutions in the D1/D2, and by 57–114 nucleotide substitutions in the ITS regions, while two strains (DMKU-PAL39 and DMKU-PAL18) were identical in both rRNA regions but differed from the type strains of the closely related species by eight nucleotide substitutions in the D1/D2, and by 10 nucleotide substitutions in the ITS regions. According to the criteria mentioned above, these strains are sufficiently separated from known species. Consequently, we were justified in assigning the two strains (DMKU-PAL186 and DMKU-PAL178) as a novel genus and species (namely, *Savitreella phatthalungensis* gen., sp. nov.) of the Taphrinomycetes (phylum Ascomycota), and two strains (DMKU-PAL39 and DMKU-PAL18) as a novel species (namely, *Goffeauzyma siamensis* sp. nov.) of the Tremellomycetes (phylum Basidiomycota). In practice, the two novel species can be distinguished from their closest related species not only by the analysis of nucleotide sequence divergence but also by phylogenetic analyses and phenotypic characteristics.

The members of *Protomyces* and *Taphrina* have been isolated from various plants, such as giant ragweed (*Ambrosia trifida*), hawk’s-beard (*Crepis japonica*), wild celery (*Apium* *nodiflorum*), the leaf gall of hedge parsley (*Torilis japonica*), Arabidopsis (*Arabidopsis thaliana*) [9,11], grey alder (*Alnus incana*), pear (*Pyrus communis*), damson plums (*Prunus insititia*), and white or silver-leaved poplars (*Populus alba*) [13]. Based on the evidence provided above, it has been suggested that the *Protomyces* and *Taphrina* species are plant-associated genera. However, there is a member of the *Protomyces* species (*P. inouyei*) that has not only been obtained from a plant but also isolated from ice in a glacier cave [36]. In addition, the members of both *Protomyces* and *Taphrina* are generally recognized as phytopathogens. Most *Protomyces* species are plant parasites causing gall symptoms in the flowers, stems, leaves, and fruits of Compositiae and Umbelliferae [9,11,37]. A large number of *Taphrina* species cause symptoms similar to those seen as a result of *Protomyces* infections, such as galls on stems, leaf curls, and witches’ brooms of various plants, especially economically important fruit trees, *viz*., peach, plum and cherry [17]. In the present study, the two strains (DMKU-PAL186^T^ and DMKU-PAL178) were isolated from healthy cultivated pineapple leaves. The pathogenicity of the strains DMKU-PAL186^T^ and DMKU-PAL178 were evaluated on pineapple leaves in a greenhouse for three weeks and the results revealed no damage or disease symptoms on the tested leaves. Thus, it could be hypothesized that these two strains might be a saprobic stage, which allows them to survive outside the host plant, or that the pineapples are non-host plants for this species. The distribution of this species may be due to human or insect transmission. In this respect, these strains (DMKU-PAL186^T^ and DMKU-PAL178) differ from the genus *Protomyces* and *Taphrina*.

The members of genus *Goffeauzyma* have been isolated from a wide range of habitats associated with acidic conditions, such as the acidic water of a volcanic environment in Argentina (*G. agrionensis*) [21], acid rock drainage in Iberian pyrite belt ecosystems in Portugal (*G. aciditolerans*, *G. iberica* and *G. metallitolerans*) [20], stomach lavage of a tuberculosis patient in Norway (*G. gastrica*) [22], soil and litter of an acidophilous beech forest in Austria (*G. gastrica*), and in soil from a tundra wetland at Cape Barrow in Alaska (*G. gilvescens*) [22]. In this study, the strains DMKU-PAL39 and DMKU-PAL18 were isolated from pineapple leaves, which were considered to be an acidic environment (pH 3–4), in Thailand. Thus, the species in genus *Goffeauzyma* appeared to occur commonly in diverse acidic environments and different geographical locations. In the samples from which the strains of the novel species were obtained, other yeast species were also isolated, namely, *Hannaella pagnoccae*, *Papiliotrema* sp., *Rhodotorula toruloides*, *Rhodosporidiobolus ruineniae*, *Symmetrospora suhii*, *Saitozyma* sp., and *Tremella* sp. This likely means that these yeast species are adapted to survive under acidic conditions. In order to evaluate differences in their genome that justify their adaptation to these environments, the genome of these new isolates should be sequenced.

## 5. Conclusions

A novel yeast genus and two novel species were described and illustrated. Based on the molecular analyses and phenotypic characteristics, the name *Savitreella phatthalungensis* gen. nov., sp. nov. is proposed in the phylum Ascomycota, and the name *Goffeauzyma siamensis* sp. nov. is proposed in the phylum Basidiomycota. In the case of *Savitreella phatthalungensis*, the pathogenicity of this species on pineapple leaves was examined, and no damage or disease symptoms were observed on the tested leaves.

## Figures and Tables

**Table 1:** Sequences of the representative species in the Taphrinomycotina (Ascomycota) used in the phylogenetic analyses.

Species	Strain	GenBank Accession Number *		
		SSU	ITS	D1/D2
*Novakomyces olei*	NCAIM Y.02187^T^	-	MW023954	MG250349
*Protomyces arabidopsidicola*	C29^T^	-	LT602858	MK934482
*Protomyces inouyei*	NRRL YB-4354^T^	AY548295	MK937056	NG_042406
*Protomyces inundatus*	NRRL Y-6349^T^	-	MK937057	U76528
*Protomyces gravidus*	NRRL Y-17093^T^	-	MK937055	U84342
*Protomyces lactucaedebilis*	NRRL YB-4353^T^	-	MK937058	U84343
*Protomyces macrosporus*	NRRL Y-12879^T^	D85143	MK937059	U94939
*Protomyces pachydermus*	NRRL YB-4355^T^	D85142	MK937060	NG_064602
*Saccharomyces cerevisiae*	CBS 1171^T^	NG_063315	AB018043	JQ689017
*Savitreella phatthalungensis*	DMKU-PAL186^T^	LC647808	MW876306	MW879743
*Savitreella phatthalungensis*	DMKU-PAL178	LC647809	MW876305	MW879742
*Saitoella complicata*	NRRL Y-17804^T^	NG_013154	JN161162	JN161156
*Saitoella coloradoensis*	CBS 12360^T^	-	KY105294	KY109521
*Schizosaccharomyces cryophilus*	CBS 11777^T^	-	NR_121468	GU470882
*Schizosaccharomyces osmophilus*	CBS 15793^T^	-	MK589403	MK253005
*Schizosaccharomyces japonicus*	CBS 354^T^	-	NR_121199	U94943
*Schizosaccharomyces pombe*	CBS 356^T^	-	MK749863	U40085
*Taphrina alni*	CBS 683.93^T^	-	AF492077	AF492024
*Taphrina antarctica*	CCFEE 5198	-	NR_132870	NG_059477
*Taphrina americana*	CBS 331.55	-	NR_155874	AF492025
*Taphrina deformans*	CBS 356.35^T^	-	MH855698	MH867217
*Taphrina dearnessii*	CBS 334.55^T^	-	MH857499	AF492037
*Taphrina betulina*	CBS 119536^T^	-	AF492080	AF492027
*Taphrina bullata*	TB004	-	KC491200	JN997390
*Taphrina carnea*	CBS 332.55^T^	-	MH857498	MH869038
*Taphrina carpini*	PYCC 5558^T^	-	NR_119488	NG_042399
*Taphrina caerulescens*	CBS 351.35^T^	-	NR_155875	NG_057691
*Taphrina confusa*	CBS 375.39^T^	-	NR_155876	NG_067349
*Taphrina communis*	CBS 352.35^T^	NG_065466	NR_160070	MH867214
*Taphrina epiphylla*	CBS 111109^T^	-	AF492096	AF492039
*Taphrina flavorubra*	CBS 377.39^T^	NG_062593	MH856045	MH867541
*Taphrina insititiae*	CBS 12782^T^	-	KC491202	JN997391
*Taphrina letifera*	CBS 335.55^T^	NG_062594	NR_155877	MH869040
*Taphrina mirabilis*	CBS 357.35^T^	NG_062595	NR_155878	MH867218
*Taphrina nana*	CBS 336.55^T^	-	MH857501	MH869041
*Taphrina padi*	CBS 693.93^T^	-	NR_155879	AF492048
*Taphrina populina*	CBS 337.55^T^	NG_062683	MH857502	NG_057694
*Taphrina populi-salicis*	CBS 419.54^T^	-	NR_155881	NG_057695
*Taphrina polystichi*	CBS 379.39^T^	-	AF492105	AF492049
*Taphrina pruni-subcordatae*	CBS 381.39^T^	NG_062596	MH856046	MH867542
*Taphrina pruni*	CBS 119537^T^	-	AF492111	MH867219
*Taphrina robinsoniana*	CBS 382.39^T^	NG_062597	NR_172369	AF492059
*Taphrina sadebeckii*	CBS 102170^T^	-	NR_155882	NG_057697
*Taphrina sacchari*	CBS 119738^T^	-	AF492117	AF492061
*Taphrina tormentillae*	CBS 339.55^T^	-	NR_155883	NG_057698
*Taphrina tosquinetii*	CBS 276.28^T^	-	NR_155884	NG_057699
*Taphrina ulmi*	CBS 420.54^T^	NG_062598	NR_155885	NG_057700
*Taphrina vestergrenii*	CBS 679.93^T^	AJ496253	NR_155886	NG_057701
*Taphrina virginica*	CBS 340.55^T^	NG_062599	AF492125	MH869044
*Taphrina veronaerambellii*	PYCC 5734^T^	-	NR_111148	NG_059911
*Taphrina wiesneri*	CBS 275.28^T^	-	NR_160065	MH866479

* Gene sequences: SSU, nuclear small subunit rRNA gene; ITS, internal transcribed spacer regions; LSU, D1/D2 domains of the nuclear large subunit rRNA gene. The newly generated sequences are in boldface. Type strains indicated by “T”.

**Table 2:** Sequences of the representative species in the Filobasidiaceae (Basidiomycota) used in the phylogenetic analyses.

Species	Strain	GenBank Accession Number *				
		SSU	ITS	D1/D2	*RPB2*	*TEF1*
*Goffeauzyma aciditolerans*	CBS 10872^T^	KF036609	NR_137808	KY107763	KF036746	KF037017
*Goffeauzyma agrionensis*	CBS 10799^T^	KF036611	NR_137809	EU627786	KF036749	KF037020
*Goffeauzyma gastrica*	CBS 2288^T^	AB032633	NR_111048	KY107764	KF036785	KF037057
*Goffeauzyma gilvescens*	CBS 7525^T^	AB032634	NR_073228	NG_058297	KF036786	KF037058
*Goffeauzyma iberica*	CBS 10871^T^	KF036636	NR_137812	NG_058298	KF036791	KF037063
*Goffeauzyma metallitolerans*	CBS 10873^T^	KF036639	NR_137813	KY107771	KF036798	KF037070
*Goffeauzyma siamensis*	DMKU-PAL39^T^	OK576181	MW669577	LC604627	LC656481	LC656484
*Goffeauzyma siamensis*	DMKU-PAL18	OK576179	MZ621116	MZ621115	LC656482	LC656483
*Filobasidium globisporum*	CBS 7642^T^	AB075546	KY103422	KY107713	KF036890	KF037153
*Filobasidium oeirense*	CBS 868^T^	KF036644	KF036644	KY103438	KY107723	KF037076
*Filobasidium uniguttulatum*	CBS 1730^T^	AB032664	AF444302	AF075468	KF036891	KF037154
*Naganishia albidosimilis*	CBS 7711^T^	KF036612	AF145325	AF137601	KF036750	KF037021
*Naganishia liquefaciens*	CBS 968^T^	KF036638	AF444345	NG_057655	KF036794	KF037066
*Naganishia globosa*	CBS 1975^T^	KF036651	AF444372	AF181540	KF036814	KF037085
*Piskurozyma capsuligena*	CBS 1906^T^	AB075544	AF444381	AF363642	KF036887	KF037152

* Gene sequences: SSU, nuclear small subunit rRNA gene; ITS, internal transcribed spacer regions; LSU, D1/D2 domains of the nuclear large subunit rRNA gene; *RPB2*, RNA polymerase II; *TEF1*, translation elongation factor 1-alpha. The newly generated sequences are in boldface. Type strains indicated by “T”.

**Table 3:** Number of nucleotide divergent and percent of nucleotide similarity in the D1/D2 domains of the LSU rRNA gene and the ITS regions between the *Savitreella phatthalungensis* sp. nov. and its closely related species.

Gene/ Species	Number of Nucleotide Substitution/Nucleotide Similarity (%)												
	*P. inouyei* NRRL YB-4354^T^	*P. pachydermus* NRRL YB-4355^T^	*P. lactucaedebilis* NRRL YB-4353^T^	*P. macrosporus* NRRL Y-12879^T^	*P. gravidus* NRRL Y-17093^T^	*P. inundatus* NRRL Y-6349^T^	*P. arabidopsidicola* C29^T^	*T. virginica* CBS 340.55^T^	*T. wiesneri* CBS 275.28^T^	*T. letifera* CBS 335.55^T^	*T. communis* CBS 352.35^T^	*T. deformans* CBS 356.35^T^	*T. carnea* CBS 332.55^T^
D1/D2	56/90.49	54/90.50	55/90.33	53/90.63	66/88.88	52/90.81	55/90.33	65/88.98	65/88.98	63/89.26	64/89.13	66/88.42	65/88.98
ITS	91/80.51	57/87.01	97/79.27	92/80.04	106/77.82	87/80.87	92/79.60	110/77.55	105/78.61	108/78.18	114/77.24	105/78.70	89/81.45

**Table 4:** Phenotypic characteristics that differentiate *Savitreella phatthalungensis* gen., sp. nov. from its closest species in the genera *Protomyces* and *Taphrina*.

Characteristics	1	2	3	4	5	6	7	8	9	10	11	12	13	14	15
Assimilation of carbon compound															
D-Galactose	-	-	v	-	+	-	-	-	w	w	-	w	-	-	-
L-Sorbose	-	-	-	-	s	-	v	-	-	-	v	-	v	v	-
*N*-Acetyl glucosamine	-	-	-	-	s	-	-	-	ND	ND	ND	ND	ND	ND	ND
D-Ribose	-	-	-	-	s	-	-	-	-	+	-	-	v	-	v
L-Arabinose	-	-	s	-	s	-	-	w	-	+	-	+	-	-	+
D-Arabinose	-	s	s	s	s	-	v	w	-	+	-	-	v	-	v
Cellobiose	s	+	s	-	s	+	v	+	+	+	+	+	v	+	+
Salicin	w	+	w	-	-	-	-	+	-	+	+	+	v	+	+
D-Gluconate	+	-	-	-	-	-	-	-	-	-	v	-	-	-	w
Assimilation of nitrogen compound															
Potassium nitrate	-	s	v	+	s	+	v	ND	w/s	+	+	+	+	+	+
Other characteristic															
Growth 25 °C	+	+	ND	-	+	ND	-	+	-	+	+	-	+	+	v
Growth 30 °C	+	ND	ND	-	ND	ND	-	-	-	-	-	-	-	-	-
Starch formation	-	w	w	w/-	w/-	w	+	ND	+	+	+	+	+	v	+

Legend of the sign: + positive; s slow positive; w weakly positive; v variable; - negative; ND not determined. Species: 1 *S. phatthalungensis*.; 2 *P. gravidus*; 3 *P. inouyei*; 4 *P. inundates*; 5 *P.* *lactucaedebilis*; 6 *P. macrosporus*; 7 *P. pachydermus*; 8 *P. arabidopsidicola*; 9 *T. vestergrenii*; 10 *T. virginica*; 11 *T. wiesneri*; 12 *T. letifera*; 13 *T. communis*; 14 *T. deformans*; 15 *T. carnea*.

**Table 5:** Phenotypic characteristics that differentiate *Goffeauzyma siamensis* sp. nov. from its closest species, *G. iberica*.

Characteristics	*G. siamensis*	*G. iberica*
Assimilation of carbon compound		
Glycerol	+	-
Ribitol	+	-
Galactitol	w	-
D-Gluconate	s	-
DL-Lactate	+	-
Citrate	-	+
Other characteristics		
0.01% Cycloheximide	-	+

Legend of the sign: + positive; s slow positive; w weakly positive; - negative.

## Data Availability

All sequence data are available in NCBI GenBank following the accession numbers in the manuscript.
